# Complete Genome Sequence of Rat Cytomegalovirus Strain ALL-03 (Malaysian Strain)

**DOI:** 10.1128/genomeA.00451-15

**Published:** 2015-06-04

**Authors:** Krishnan Nair Balakrishnan, Ashwaq Ahmed Abdullah, Siti Nazrina Camalxaman, Yi Wan Quah, Yusuf Abba, Homayoun Hani, Hwei San Loh, Farina Mustaffa Kamal, Nazariah Allaudin Zeenathul, Ideris Aini, A. R. Omar, Mohamed Mustapha Noordin, Mohd Lila Mohd Azmi

**Affiliations:** a Department of Pathology and Microbiology, Faculty of Veterinary Medicine, Universiti Putra Malaysia, Serdang, Selangor, Malaysia; b Institute of Bioscience, Universiti Putra Malaysia, Serdang, Selangor, Malaysia; c School of Biosciences, The University of Nottingham, Semenyih, Selangor, Malaysia; d Department of Medical Laboratory Technology, Faculty of Health Sciences, Universiti Teknologi MARA, Selangor, Malaysia

## Abstract

The complete genome sequence of the ALL-03 strain of rat cytomegalovirus (RCMV) has been determined. The RCMV genome has a length of 197,958 bp and is arranged as a single unique sequence flanked by 504-bp terminal direct repeats. This strain is closely related to the English strain of RCMV in terms of genetic arrangement but differs slightly in size.

## GENOME ANNOUNCEMENT

Cytomegalovirus (CMV) belongs to the *Betaherpesvirus* subfamily of herpesvirus (1). Since CMV is species specific, there is the need for a suitable model for human CMV (HCMV) infection and pathogenicity; hence, a rat model became the choice for study. Understanding the organization of genomic content is very vital to understanding the mechanisms of infection in an RCMV rat model. Thus, through an arrangement of the DNA sequence obtained, we discovered it was novel and belonged to the Malaysian strain of RCMV, ALL-03. This strain is the third RCMV for which the complete genome sequence has been generated after the RCMV-Maastricht and RCMV-English strains. The Maastricht strain (RCMV-M) was the first isolation portrayed by Bruggeman et al. in 1982 (2), and around the same year, Priscott and Tyrrell (3) reported the presence of the English isolate. The complete genome sequences available for RCMV English strain (4) and Maastricht strain (5) had genome lengths of 202,946 bp and 229,896 bp, respectively. Interestingly, both of these viruses were isolated from the *Rattus norvegicus* but are classified under different species within betaherpesvirus instead of diverse strains of the same RCMV. This classification was based on significant differences in restriction enzyme cleavage patterns (4, 6). Conversely, RCMV ALL-03 was isolated from the placenta and uterus of *Rattus rattus diardii* (house rat) (7), and it is unique in the sense that it can cause vertical transmission, infecting the offspring (8).

RCMV ALL-03 was propagated in a rat embryo fibroblast (REF) cell line, and the genomic DNA was extracted with high concentration and purity to analyze the viral genome by sequencing using Illumina sequencing technology. The raw data generated from the Genome Analyzer IIX were converted to readable sequences, and the subsequent information was inserted into CLC Genomics Workbench version 4.7.2 to analyze the sequence data. The trimmed sequences were assembled to form a set of contigs, and the areas of similarity within the contigs were distinguished utilizing Basic Local Alignment Search Tool (BLAST).

Overall, a total 197,958 bp of genome length with a 46% G+C content was obtained from the complete genome sequence of RCMV ALL-03. From this, a total of 123 protein-coding genes (CDSs) were identified, 36.2% (46 CDSs) of which have been annotated as genes and arranged into 8 functional classes, such as capsid, tegument, glycoprotein, DNA replication, DNA packaging, nuclear egress, immune evasion, and regulatory. RCMV ALL-03 CDSs were found to have higher homology with RCMV-E (MuHV-8), in which BLASTx analysis indicated approximately 99% similarity with MuHV-8 compared to MuHV-2 and MuHV-1. Besides that, there were 111 and 107 CDSs homologous to MuHV-1 and MuHV-2, respectively. The above-mentioned data suggest that RCMV ALL-03 is the closest to MuHV-8, with an estimated 8.2 substitutions per 1 kbp difference from MuHV-8. This clearly shows that RCMV ALL-03 belongs to the same species of MuHV-8 but a different strain.

### Nucleotide sequence accession number. 

The complete genome sequence of RCMV ALL-03 has been deposited at NCBI under the accession no. KP967684.
